# 1,3-Bis(bromo­meth­yl)-2-nitro­benzene

**DOI:** 10.1107/S160053681000718X

**Published:** 2010-03-13

**Authors:** Muhammad Nadeem Arshad, Katheryne Zumberge Edson, Scott T. Mough, K. Travis Holman

**Affiliations:** a Department of Chemistry, GC University Lahore 54000, Pakistan; b Department of Chemistry, Georgetown University, 37th and O St NW, Washington, DC 20057, USA

## Abstract

In the title compound, C_8_H_7_Br_2_NO_2_, an inter­mediate for the synthesis of macrocycles, the NO_2_ group makes a dihedral angle of 65.07 (19)° with the arene ring, and the bromo­methyl substituents adopt a *trans* conformation about the ring such that the mol­ecule closely approximates *C*2 symmetry.

## Related literature

For related structures, see: Li *et al.* (2006 ▶); Qin *et al.* (2005 ▶). For related compounds, see: Raatikainen *et al.* (2008 ▶); Mough *et al.* (2004 ▶). For the synthesis, see: Boeckmann & Vögtle (1981 ▶). 
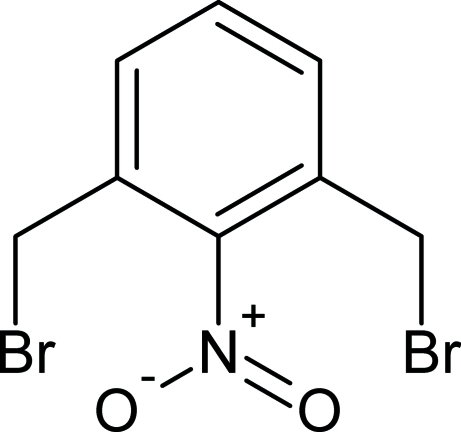


## Experimental

### 

#### Crystal data

C_8_H_7_Br_2_NO_2_
*M* *_r_* = 308.97
Monoclinic, 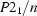

*a* = 7.7837 (13) Å
*b* = 7.7573 (13) Å
*c* = 15.938 (3) Å
β = 90.933 (3)°
*V* = 962.2 (3) Å^3^
*Z* = 4
Mo *K*α radiation
μ = 8.39 mm^−1^
*T* = 185 K
0.20 × 0.20 × 0.08 mm

#### Data collection

Bruker SMART 1K diffractometer
Absorption correction: multi-scan (*SADABS*; Bruker, 2001 ▶) *T*_min_ = 0.285, *T*_max_ = 0.553
8228 measured reflections
2259 independent reflections
1812 reflections with *I* > 2σ(*I*)
*R*_int_ = 0.041

#### Refinement

*R*[*F* ^2^ > 2σ(*F* ^2^)] = 0.037
*wR*(*F* ^2^) = 0.083
*S* = 1.06
2259 reflections
118 parameters
H-atom parameters constrained
Δρ_max_ = 1.35 e Å^−3^
Δρ_min_ = −1.31 e Å^−3^

### 

Data collection: *SMART* (Bruker, 2001 ▶); cell refinement: *SAINT* (Bruker, 2001 ▶); data reduction: *SAINT*; program(s) used to solve structure: *SHELXS97* (Sheldrick, 2008 ▶); program(s) used to refine structure: *SHELXL97* (Sheldrick, 2008 ▶); molecular graphics: *ORTEP-3* (Farrugia, 1997 ▶), *PLATON* (Spek, 2009 ▶) and *X-SEED* (Barbour, 2001 ▶); software used to prepare material for publication: *WinGX* (Farrugia, 1999 ▶) and *PLATON*.

## Supplementary Material

Crystal structure: contains datablocks global, I. DOI: 10.1107/S160053681000718X/ng2729sup1.cif

Structure factors: contains datablocks I. DOI: 10.1107/S160053681000718X/ng2729Isup2.hkl

Additional supplementary materials:  crystallographic information; 3D view; checkCIF report

## Figures and Tables

**Fig. 1.**
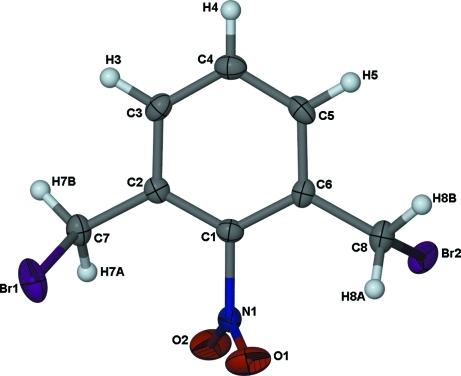
The labelled thermal ellipsoid plot of (I) at the 50% probability level.

**Fig. 2.**
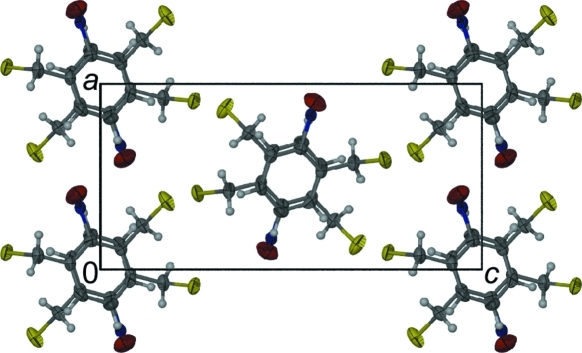
The unit cell packing diagram of (I) as viewed down the b axis.

## supplementary crystallographic information

### Comment

Derivatives of 1,3-bis(bromomethyl)benzene have been widely used to synthesize macrocycles via S_N_2 reactions. For recent examples, see Raatikainen *et al.*, 2008 and Mough *et al.*, 2004.

The crystal structure of the title compound is in comparison with the already reported 1,3-bis(bromomethyl)benzene (II) (Li *et al.*, 2006) and 2,3-bis(bromomethyl)-1-methoxy-4-nitrobenzene (III) (Qin *et al.*, 2005). The nitro group of I is oriented at a dihedral angle of 65.07(0.19)° to the arene ring. The bromomethyl groups (C7/Br1 and C8/Br2) are oriented anti to each other and exhibit almost identical dihedral angles with respect to arene ring carbons atoms (80.34(0.27)° and 80.99(0.28)°, respectively). The molecules therefore closely approximate *C*2 point group symmetry in the crystal.

### Experimental

The title compound was prepared following the method reported by Boeckmann and Vögtle *et al.*, 1981.

### Refinement

The aromatic and methylene H-atoms were positioned geometrically and refined using a riding model with d(C—H) = 0.95 Å, U_iso_=1.2U_eq_ (C) for aromatic 0.99 Å, U_iso_ = 1.2U_eq_ (C) for methylene

Electron density synthesis with coefficients Fo—Fc: Highest peak 1.35 at 0.7472 0.2174 0.6981 [ 0.78 A from BR2 ] Deepest hole -1.31 at 0.7372 0.0534 0.6462 [ 0.75 A from BR2 ]

### Crystal data

**Table:**

C_8_H_7_Br_2_NO_2_	*F*(000) = 592
*M**_r_* = 308.97	*D*_x_ = 2.133 Mg m^−^^3^
Monoclinic, *P*2_1_/*n*	Mo *K*α radiation, λ = 0.71073 Å
Hall symbol: -P 2yn	Cell parameters from 1024 reflections
*a* = 7.7837 (13) Å	θ = 2.6–28.0°
*b* = 7.7573 (13) Å	µ = 8.39 mm^−^^1^
*c* = 15.938 (3) Å	*T* = 185 K
β = 90.933 (3)°	Needles, colorless
*V* = 962.2 (3) Å^3^	0.20 × 0.20 × 0.08 mm
*Z* = 4	

### Data collection

**Table:**

Bruker SMART 1K diffractometer	2259 independent reflections
Radiation source: fine-focus sealed tube	1812 reflections with *I* > 2σ(*I*)
graphite	*R*_int_ = 0.041
ω scan	θ_max_ = 28.0°, θ_min_ = 2.6°
Absorption correction: multi-scan (*SADABS*; Bruker, 2001)	*h* = −10→10
*T*_min_ = 0.285, *T*_max_ = 0.553	*k* = −10→10
8228 measured reflections	*l* = −21→20

### Refinement

**Table:**

Refinement on *F*^2^	Primary atom site location: structure-invariant direct methods
Least-squares matrix: full	Secondary atom site location: difference Fourier map
*R*[*F*^2^ > 2σ(*F*^2^)] = 0.037	Hydrogen site location: inferred from neighbouring sites
*wR*(*F*^2^) = 0.083	H-atom parameters constrained
*S* = 1.06	*w* = 1/[σ^2^(*F*_o_^2^) + (0.0289*P*)^2^ + 1.9462*P*] where *P* = (*F*_o_^2^ + 2*F*_c_^2^)/3
2259 reflections	(Δ/σ)_max_ < 0.001
118 parameters	Δρ_max_ = 1.35 e Å^−^^3^
0 restraints	Δρ_min_ = −1.31 e Å^−^^3^

### Special details

**Table:**

Geometry. All esds (except the esd in the dihedral angle between two l.s. planes) are estimated using the full covariance matrix. The cell esds are taken into account individually in the estimation of esds in distances, angles and torsion angles; correlations between esds in cell parameters are only used when they are defined by crystal symmetry. An approximate (isotropic) treatment of cell esds is used for estimating esds involving l.s. planes.
Refinement. Refinement of *F*^2^ against ALL reflections. The weighted *R*-factor wR and goodness of fit *S* are based on *F*^2^, conventional *R*-factors *R* are based on *F*, with *F* set to zero for negative *F*^2^. The threshold expression of *F*^2^ > σ(*F*^2^) is used only for calculating *R*-factors(gt) etc. and is not relevant to the choice of reflections for refinement. *R*-factors based on *F*^2^ are statistically about twice as large as those based on *F*, and *R*- factors based on ALL data will be even larger.

### Fractional atomic coordinates and isotropic or equivalent isotropic displacement parameters (Å^2^)

**Table:**

	*x*	*y*	*z*	*U*_iso_*/*U*_eq_	
Br1	0.20766 (5)	0.59088 (5)	0.74548 (2)	0.03326 (13)	
Br2	0.26732 (6)	0.86865 (6)	0.32665 (3)	0.04186 (15)	
C1	0.2474 (4)	0.6504 (4)	0.5246 (2)	0.0211 (7)	
N1	0.2391 (4)	0.8324 (4)	0.55227 (19)	0.0245 (7)	
C2	0.1776 (4)	0.6087 (4)	0.4461 (2)	0.0203 (7)	
C3	0.1891 (5)	0.4378 (5)	0.4212 (2)	0.0238 (8)	
H3	0.1450	0.4051	0.3676	0.029*	
O1	0.3753 (4)	0.9089 (4)	0.5617 (2)	0.0434 (8)	
C6	0.3228 (4)	0.5301 (5)	0.5786 (2)	0.0205 (7)	
C5	0.3295 (5)	0.3604 (5)	0.5504 (2)	0.0249 (8)	
H5	0.3807	0.2750	0.5855	0.030*	
C7	0.3935 (5)	0.5748 (5)	0.6637 (2)	0.0281 (8)	
H7A	0.4550	0.6864	0.6610	0.034*	
H7B	0.4768	0.4854	0.6820	0.034*	
O2	0.0993 (4)	0.8953 (4)	0.5634 (2)	0.0426 (8)	
C4	0.2638 (5)	0.3131 (5)	0.4729 (2)	0.0260 (8)	
H4	0.2695	0.1965	0.4550	0.031*	
C8	0.0943 (5)	0.7371 (5)	0.3889 (2)	0.0259 (8)	
H8A	0.0239	0.8174	0.4222	0.031*	
H8B	0.0170	0.6766	0.3487	0.031*	

### Atomic displacement parameters (Å^2^)

**Table:**

	*U*^11^	*U*^22^	*U*^33^	*U*^12^	*U*^13^	*U*^23^
Br1	0.0391 (2)	0.0396 (2)	0.0212 (2)	−0.00245 (18)	0.00494 (16)	−0.00610 (16)
Br2	0.0346 (2)	0.0541 (3)	0.0370 (3)	−0.0017 (2)	0.00213 (18)	0.0232 (2)
C1	0.0198 (17)	0.0187 (17)	0.0247 (18)	−0.0006 (14)	0.0027 (14)	−0.0005 (14)
N1	0.0324 (18)	0.0223 (16)	0.0188 (15)	0.0027 (13)	−0.0022 (13)	0.0000 (12)
C2	0.0161 (16)	0.0231 (18)	0.0218 (18)	−0.0003 (14)	0.0033 (14)	0.0018 (14)
C3	0.0227 (18)	0.030 (2)	0.0190 (18)	−0.0043 (15)	0.0030 (15)	−0.0041 (15)
O1	0.0381 (17)	0.0343 (16)	0.058 (2)	−0.0126 (14)	0.0095 (15)	−0.0141 (15)
C6	0.0153 (16)	0.0287 (18)	0.0175 (17)	−0.0005 (14)	0.0020 (14)	0.0005 (14)
C5	0.0254 (19)	0.0218 (17)	0.028 (2)	0.0038 (15)	0.0060 (16)	0.0076 (15)
C7	0.0247 (19)	0.038 (2)	0.0215 (19)	0.0002 (16)	−0.0013 (15)	0.0014 (16)
O2	0.0310 (16)	0.0359 (16)	0.061 (2)	0.0146 (13)	−0.0064 (15)	−0.0158 (15)
C4	0.0258 (19)	0.0215 (18)	0.031 (2)	−0.0022 (15)	0.0074 (16)	−0.0021 (15)
C8	0.0228 (19)	0.031 (2)	0.0236 (19)	−0.0007 (16)	0.0001 (15)	0.0058 (15)

### Geometric parameters (Å, °)

**Table:**

Br1—C7	1.967 (4)	C3—H3	0.9500
Br2—C8	1.971 (4)	C6—C5	1.392 (5)
C1—C6	1.392 (5)	C6—C7	1.497 (5)
C1—C2	1.395 (5)	C5—C4	1.379 (5)
C1—N1	1.480 (5)	C5—H5	0.9500
N1—O2	1.209 (4)	C7—H7A	0.9900
N1—O1	1.222 (4)	C7—H7B	0.9900
C2—C3	1.387 (5)	C4—H4	0.9500
C2—C8	1.491 (5)	C8—H8A	0.9900
C3—C4	1.392 (5)	C8—H8B	0.9900
			
C6—C1—C2	123.6 (3)	C6—C5—H5	119.2
C6—C1—N1	118.4 (3)	C6—C7—Br1	110.6 (2)
C2—C1—N1	118.0 (3)	C6—C7—H7A	109.5
O2—N1—O1	124.5 (3)	Br1—C7—H7A	109.5
O2—N1—C1	118.2 (3)	C6—C7—H7B	109.5
O1—N1—C1	117.3 (3)	Br1—C7—H7B	109.5
C3—C2—C1	116.9 (3)	H7A—C7—H7B	108.1
C3—C2—C8	119.6 (3)	C5—C4—C3	119.4 (3)
C1—C2—C8	123.5 (3)	C5—C4—H4	120.3
C2—C3—C4	121.6 (3)	C3—C4—H4	120.3
C2—C3—H3	119.2	C2—C8—Br2	111.1 (2)
C4—C3—H3	119.2	C2—C8—H8A	109.4
C1—C6—C5	116.9 (3)	Br2—C8—H8A	109.4
C1—C6—C7	123.3 (3)	C2—C8—H8B	109.4
C5—C6—C7	119.7 (3)	Br2—C8—H8B	109.4
C4—C5—C6	121.7 (3)	H8A—C8—H8B	108.0
C4—C5—H5	119.2		
			
C6—C1—N1—O2	−115.0 (4)	N1—C1—C6—C5	−179.6 (3)
C2—C1—N1—O2	64.5 (5)	C2—C1—C6—C7	−178.4 (3)
C6—C1—N1—O1	65.5 (5)	N1—C1—C6—C7	1.0 (5)
C2—C1—N1—O1	−115.1 (4)	C1—C6—C5—C4	−0.3 (5)
C6—C1—C2—C3	−1.5 (5)	C7—C6—C5—C4	179.1 (3)
N1—C1—C2—C3	179.1 (3)	C1—C6—C7—Br1	80.1 (4)
C6—C1—C2—C8	179.0 (3)	C5—C6—C7—Br1	−99.3 (3)
N1—C1—C2—C8	−0.4 (5)	C6—C5—C4—C3	0.2 (5)
C1—C2—C3—C4	1.2 (5)	C2—C3—C4—C5	−0.6 (5)
C8—C2—C3—C4	−179.2 (3)	C3—C2—C8—Br2	−98.9 (3)
C2—C1—C6—C5	1.0 (5)	C1—C2—C8—Br2	80.6 (4)
